# Supplementary material for: Human Assembloid Model of Emergent Neurotropic Enteroviruses
Source: bioRxiv. 2025 Nov 19:2025.11.18.689148. Preprint. [Version 1] doi: 10.1101/2025.11.18.689148 (PMC12667899; doi:10.1101/2025.11.18.689148)
Supplement: Supplement 6 [file NIHPP2025.11.18.689148v1-supplement-6.pdf]

bioRxiv preprint doi: https://doi.org/10.1101/2025.11.18.689148; this version posted November 19, 2025. The copyright holder for this preprint (which was not certified by peer review) is the author/funder, who has granted bioRxiv a license to display the preprint in perpetuity. It is made available under aCC-BY 4.0 International license.

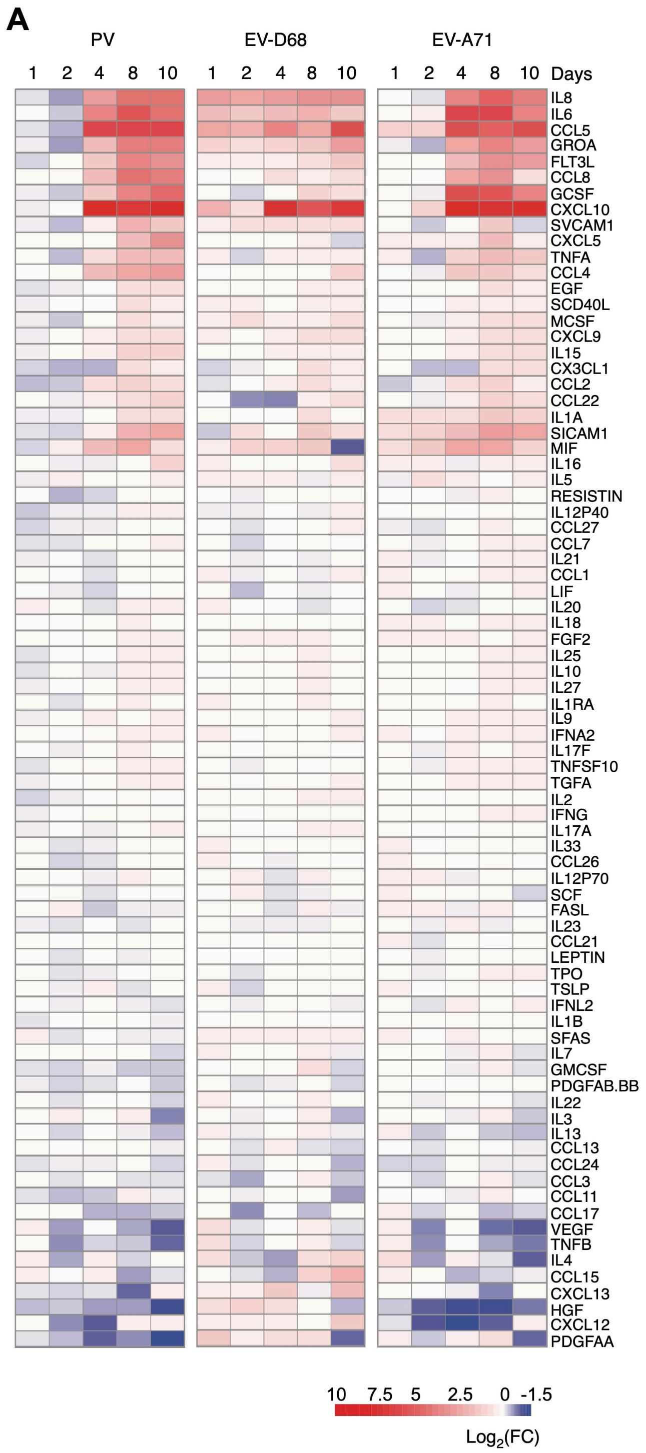

Supplemental Figure 1: Cytokine profiling of EV infected hSpO, Related to Figure 2. (A) Luminex-based multianalyte profiling of 80 cytokines and chemokines of supernatants from EV infected hSpO at indicated days post infection. Cytokines are graphed as log2 fold change of mean fluorescent intensity (MFI) over uninfected controls at matched timepoint. MFI is the average of two technical replicates. Red denotes increased cytokines in comparison to uninfected, blue denotes decreased cytokines compared to uninfected and white denotes little to no change.

bioRxiv preprint doi: https://doi.org/10.1101/2025.11.18.689148; this version posted November 19, 2025. The copyright holder for this preprint (which was not certified by peer review) is the author/funder, who has granted bioRxiv a license to display the preprint in perpetuity. It is made available under aCC-BY 4.0 International license.

A

Total number of cells per run

Run	Line	Uninfected	PV	EV–D68	EV–A71
1	8119-1	6,072	5,906	5,355	6,939
2	8119-1	8,110	-	9,254	7,078
3	8119-1	7,045	-	-	6,843
4	8119-1	4,866	14,899	-	-
4	0524-1	8,901	6,473	-	8,736

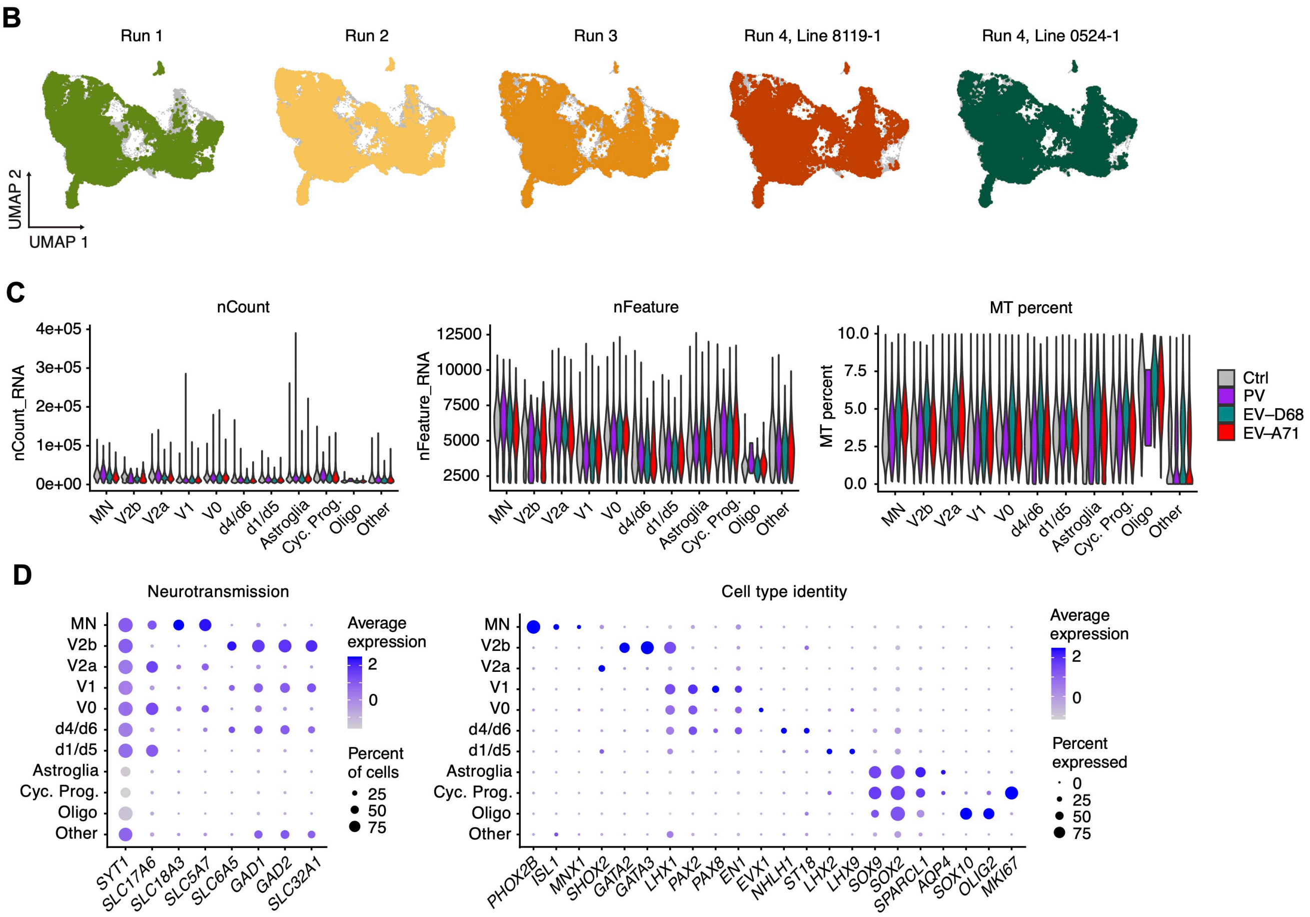

Supplemental Figure 2: Quality metrics of single cell sequencing runs, Related to Figure 3. (A) hiPS cell lines used in each single cell run. Total number of cells from each enterovirus infected sample or uninfected sample per single cell run. **(B)** UMAP plots showing cells separately colored by the single cell run they were derived from. **(C)** Violin plots of total RNA count, gene count and mitochondrial gene content per each cell type cluster across uninfected and infected samples. **(D)** Dot plots showing the expression of selected neurotransmitter and cell-specific identity related genes. The size of the circle represents the percent of cells expressing each gene per cluster.

bioRxiv preprint doi: https://doi.org/10.1101/2025.11.18.689148; this version posted November 19, 2025. The copyright holder for this preprint (which was not certified by peer review) is the author/funder, who has granted bioRxiv a license to display the preprint in perpetuity. It is made available under aCC-BY 4.0 International license.

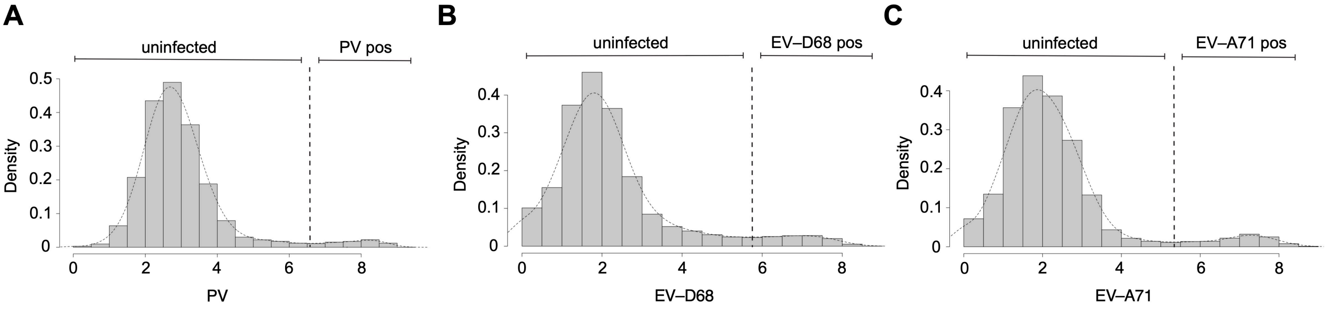

D

Number of infected cells per run

Run	hiPSC line	PV	% Infected	EV–D68	% Infected	EV–A71	% Infected
1	8119-1	378	6.40	221	4.13	371	5.35
2	8119-1	-	-	637	6.88	449	6.34
3	8119-1	-	-	-	-	329	4.81
4	8119-1	400	2.68	-	-	-	-
4	0524-1	272	4.20	-	-	611	5.07

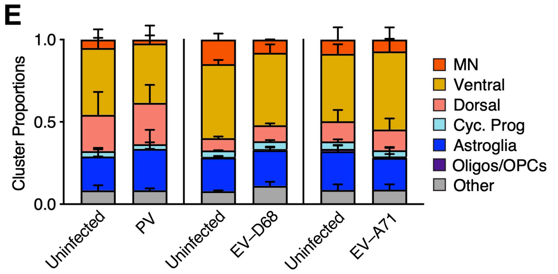

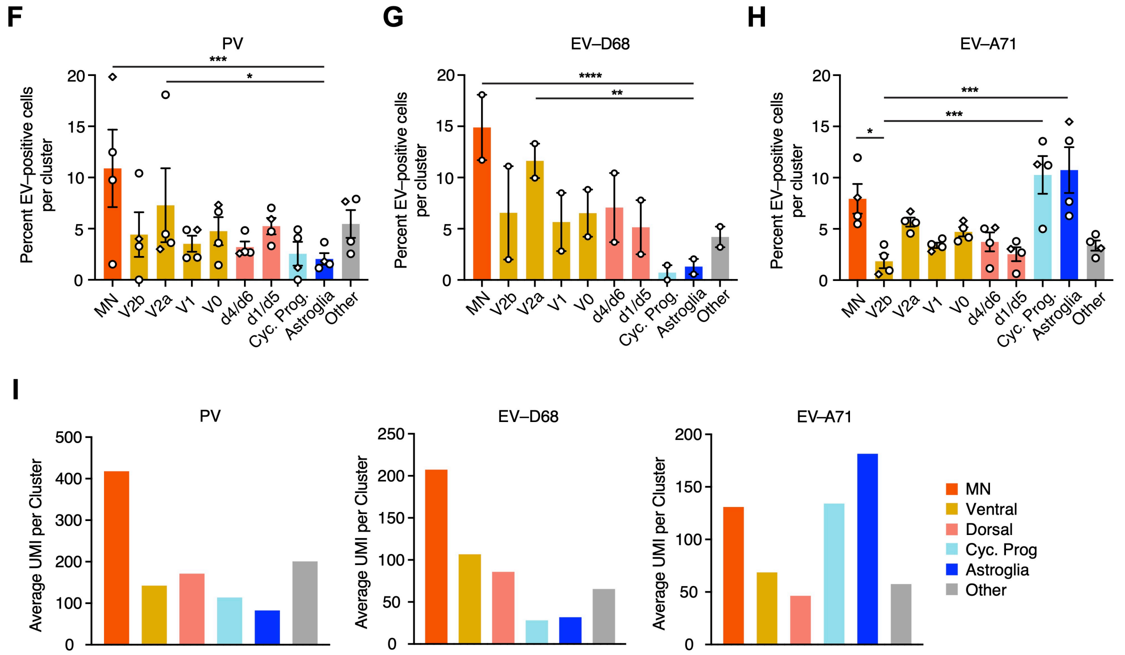

Supplemental Figure 3: Identification of enterovirus infected cells by single cell sequencing, Related to Figure 3. (A-C) Histograms of virus expression for PV, EV-D68 and EV-A71 samples in scRNA-seq experiment. Histograms show bimodal distribution of virus expression. Dotted line indicates set threshold to discriminate between infected cells supporting active virus replication versus those containing viral RNA due to other processes (uninfected cells). **(D)** Distribution of the number of infected cells across each single cell run. **(E)** Graphs showing the percentage of cells from each sample belonging to each cluster. Each virus infected condition is matched to the uninfected samples from the same single cell runs. Datasets represent mean ± SD. **(F-H)** Percentage of infected cells per cell type in PV, EV-D68 and EV-A71 samples as graphed in Figure 3 separated by individual cell cluster. Datasets represent mean ± SEM. P-values were determined by two-way ANOVA with Benjamini and Hochberg correction. *P<0.05, **P<0.01, ***P<0.001, ****P<0.0001. **(I)** Average of the raw UMI counts of indicated enterovirus per cell type across all cells in PV, EV-D68 and EV-A71 infected samples.

bioRxiv preprint doi: https://doi.org/10.1101/2025.11.18.689148; this version posted November 19, 2025. The copyright holder for this preprint (which was not certified by peer review) is the author/funder, who has granted bioRxiv a license to display the preprint in perpetuity. It is made available under aCC-BY 4.0 International license.

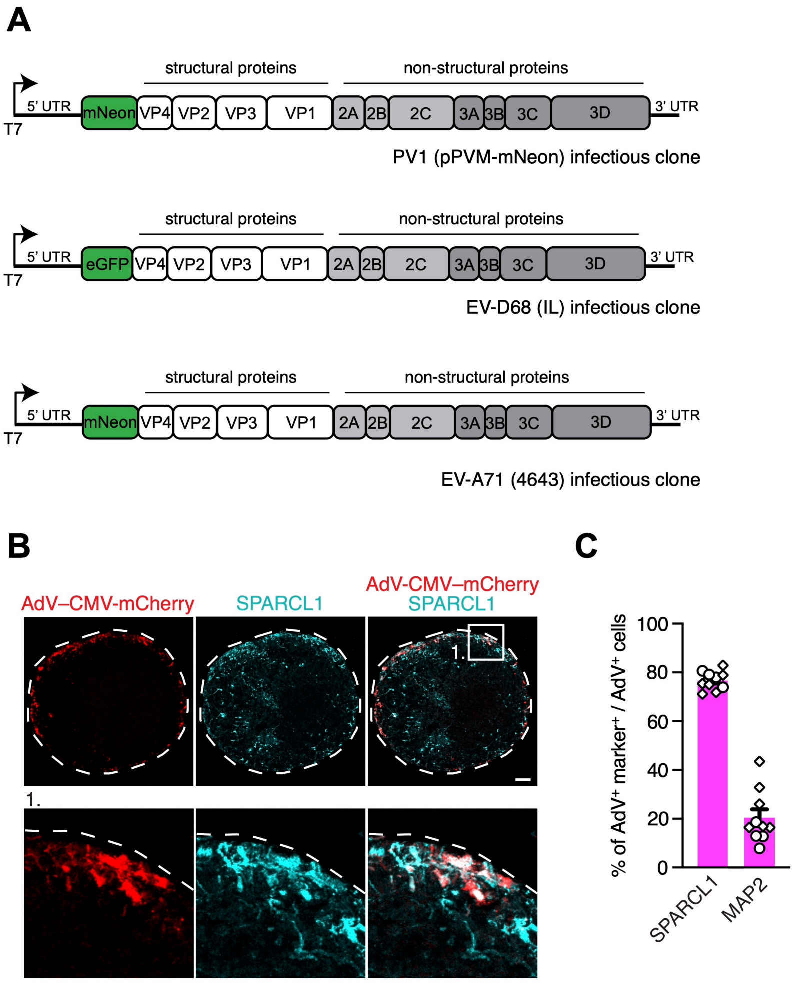

Supplemental Figure 4: Validation of live imaging with reporter viruses, Related to Figure 4. (A) Schematic depiction of the PV1 infectious clone expressing mNeon with a GPI anchor tag, the EV-D68 (IL) infectious clone expressing eGFP, and the EV-A71 (4643) infectious clone expressing mNeon. T7, T7 promoter, UTR, untranslated region. **(B)** Representative SPARCL1 immunostainings of hSpO infected with AdV-CMV-mCherry. Scale bar, 100 µm. **(C)** Quantifications of AdV-CMV-mCherry co-localization with the neuronal maker MAP2 or glial marker SPARCL1. n= 10 hSpO from 2 hiPS cell lines from 1 differentiation. Datasets represent mean ± SEM.

bioRxiv preprint doi: https://doi.org/10.1101/2025.11.18.689148; this version posted November 19, 2025. The copyright holder for this preprint (which was not certified by peer review) is the author/funder, who has granted bioRxiv a license to display the preprint in perpetuity. It is made available under aCC-BY 4.0 International license.

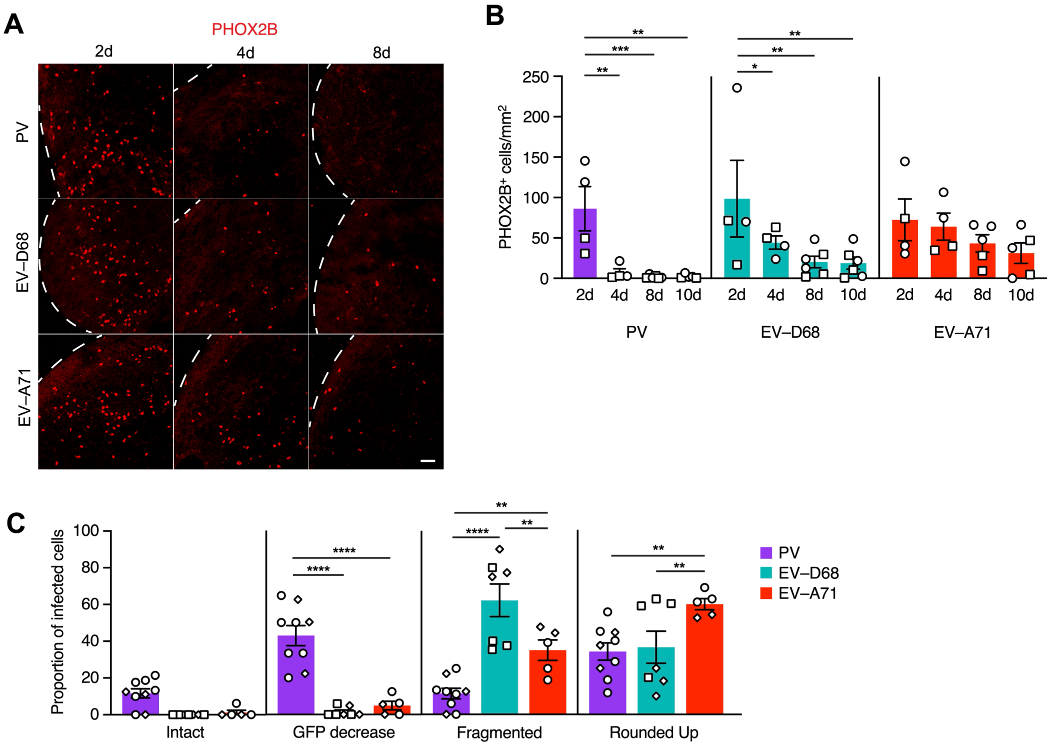

Supplemental Figure 5: Cell damage in EV-infected hSpO, Related to Figure 5. (A) Representative PHOX2B immunostainings of hSpO infected with PV, EV-D68, or EV-A71 at indicated days post infection. Scale bar, 50 µm. **(B)** Number of PHOX2B positive cells per hSpO area for PV, EV-D68, or EV-A71 infected hSpO. n= 19 PV infected hSpO, n= 20 EV-D68 infected hSpO, n= 18 EV-A71 infected hSpO from 2 hiPS cell lines from 2 differentiations. Values represent mean ± SEM. P-values were determined by two-way ANOVA (adjusted with Benjamini–Hochberg). *P<0.05, **P<0.01, ****P<0.0001. **(C)** Damage bins as related to Figure 5B, with individual values graphed. n= 9 PV infected hSpO from 2 hiPS cell lines from 1 differentiation, n= 7 EV-D68 infected hSpO from 2 hiPS cell lines from 2 differentiations, n= 5 EV-A71 infected hSpO from 2 hiPS cell lines from 2 differentiations. Datasets represent mean ± SEM. P-values were determined by two-way ANOVA (adjusted with Benjamini–Hochberg). **P<0.01, ****P<0.0001.

Supplemental Video 1. Spontaneous muscle contractions in uninfected hSpO-hSkM.
Supplemental Video 2. Spontaneous muscle contractions in PV infected hSpO-hSkM.
Supplemental Video 3. Spontaneous muscle contractions in EV-D68 infected hSpO-hSkM.
Supplemental Video 4. Spontaneous muscle contractions in EV-A71 infected hSpO-hSkM.
